# Effect of intravenous iron on endogenous erythropoietin and FGF-23 secretion in patients with chronic kidney disease

**DOI:** 10.1080/0886022X.2022.2164305

**Published:** 2023-01-23

**Authors:** Katarzyna Muras-Szwedziak, Ewa Pawłowicz-Szlarska, Michał Nowicki

**Affiliations:** Department of Nephrology, Hypertension and Kidney Transplantation, Medical University of Lodz, Lodz, Poland

**Keywords:** Anemia, iron, erythropoietin, fibroblast growth factor-23, inflammation

## Abstract

**Introduction:**

It has been observed that intravenous iron administration may suppress endogenous production of erythropoietin (EPO). We postulate that this effect may be mediated by increased FGF-23 secretion.

**Aim of the study:**

To evaluate the short-term effect of intravenous iron sucrose administration on endogenous EPO secretion in patients with chronic kidney disease (CKD).

**Materials and methods:**

The cohort comprised 35 nondialysis patients with CKD stages 3–5. All received 100 mg of intravenous iron (III)-hydroxide sucrose complex daily for five consecutive days. Plasma EPO, iFGF-23, cFGF-23, PTH, bone alkaline phosphatase (BAP), phosphorus (PO4), calcium (Ca), and high-sensitive C-reactive protein (CRP) were measured before, and two hours after, the first and third iron infusions, and after completing iron therapy.

**Results:**

EPO concentration at the end of iron treatment was significantly lower than two hours after the first iron infusion (*p* = 0.0003) and before the third dose (*p* = 0.0006) (12.6 [10.2, 41.4] mIU/mL. vs. 30.9 [15.9, 54.2] mIU/mL and 33.4 [15.4, 56.7] mIU/mL, respectively). Conversely, plasma iFGF-23 was significantly higher before the third dose (61.1 [18.6, 420.1 4] pg/mL; *p* = 0.025) and after the course of treatment (92.1 [28.4, 878.1] pg/mL; *p* = 0.004) compared to pretreatment value (48.4 [16.2, 420] pg/mL). cFGF-23 concentration was significantly lower than baseline after the first iron dose (491.8 [257.7, 1086.3] vs. 339.2 [75.4, 951.2] RU/mL; *p* = 0.005) and after treatment (398.7 [90.4, 1022.3] RU/mL; *p* = 0.025). No significant linear correlation was found between changes in plasma EPO and FGF-23.

**Conclusions:**

Although intravenous iron therapy causes parallel increase of FGF-23 and supression of endogenous EPO, these two effects seem to be independent.

## Introduction

The most common long-term complications of chronic kidney disease (CKD) are anemia and mineral metabolism disorders [1]. Both often coexist and are interrelated [1,2]

The two main causes of renal anemia are relative erythropoietin deficiency and concomitant functional iron deficiency. Hence, for more than 30 years, the correction of iron metabolism disorders has formed the basis of renal anemia treatment by using intravenous iron preparations administered with agents that stimulate erythropoiesis [3].

In patients with severe iron deficiency and advanced chronic kidney disease, the intravenous route of iron administration is preferred. However, a risk of adverse reactions such as anaphylaxis, edema, local reactions at the puncture site and chronic complications, such as pro-inflammatory effects, oxidative stress or phosphate metabolism disorders, needs to be considered, even through they are rare nowadays [4].

Although its clinical relevance has not been sufficiently established, intravenous iron infusion may have a potential direct, or indirect, suppressive effect on endogenous erythropoietin secretion, and this needs to be taken into account in the current renal anemia treatment regimen [5]. It has been postulated that iron-mediated changes in mineral metabolism may exacerbate existing renal anemia due to the proinflammatory response to iron infusions [4,5].

Intravenous iron supplementation has been found to cause significant dysregulation of calcium and phosphate metabolism mediated by increased secretion of FGF23, thus resulting in hypophosphatemia [6]. Furthermore, a single experimental study showed that intravenous iron supplementation increased FGF23 plasma level while decreasing serum endogenous erythropoietin concentration [7]. It has been also postulated that this effect could be mediated by the induction of inflammation, which may thereby disturb erythropoiesis [8]. Such effects were also reported in FGF23-deficient mouse (Fgf23^2/2^) in which low FGF23 activity attenuated inflammation, resulting in increased serum iron and ferritin levels and correction in anemia [9].

It was also found that the administration of recombinant human erythropoietin increased the removal of iFGF23, resulting in a decrease of iFGF23 to cFGF23 ratio, which inhibits the adverse effects of iFGF23 on erythropoiesis and erythropoietin production [10]. Hiram-Bab et al. [11] showed that mice overexpressing the EPO coding gene developed severe osteopenia. In another study, administration of recombinant erythropoietin reduced bone density and increased bone remodeling [12].

The mechanism of this effect has been unclear but may be facilitated by the activated progenitor cells of the erythrocyte line that express FGF23 receptors [11,12].

To explore these complex relations in the clinical settings, the present clinical study evaluates the short-term effect of intravenous iron sucrose treatment on endogenous EPO secretion in patients with chronic kidney disease.

## Materials and methods

This exploratory prospective single-arm study included 35 patients with CKD stage 3–5, enrolled in a single reference nephrology center. None of the patients (24 women and 11 men; mean age 60.1±12.3 years) were on dialysis. Their mean estimated glomerular filtration rate (eGFR) was 22.9 ± 10.4 mL/min per 1.73 m^2^.

The baseline characteristics of the study group are provided in Table 1. The study protocol was approved by the Local Ethics Committee and written informed consent was obtained from all participants (Number of ethical approval – RNN/307/13/KE).

**Table 1.** Baseline clinical and biochemical parameters in the study group.

Parameter	Value
Females/Males	24/11
Age [years]	60.1 ± 12.3
Hemoglobin [g/dL]	9.57 ± 1.31
Iron [μg/dL]	15.9 ± 8.43
TSAT [%]	26.34 ± 10.76
Ferritin [mg/mL]	211 [160, 375]
CRP [mg/L]	6.5 [4.3, 15.2]
Ca [mmol/L]	2.0 ± 0.26
Phosphate [mmol/L]	1.59 [1.35, 1.8]
iFGF-23 [pg/mL]	48.4 [16.2, 420]
cFGF-23 [RU/mL]	491.8 [257.7, 1086.3]
EPO [mIU/mL]	16.7 [12.6, 54.3]
PTH [pg/mL]	3.51 [1.2, 21.08]
BAP [U/L]	22.9 [19.3, 27.4]
Creatinine [mg/dL]	3.39 [2.18, 5.29]
eGFR [mL/min/1.73 m^2^]	22.9 ± 10.4
Body mass [kg]	68 [62, 73]
BMI [kg/m^2^]	23.78 [22.06, 24.8]

Data presented as mean ± SD or median [Q1, Q3] according to the variable distribution. TSAT: transferrin saturation; CRP: C-reactive protein; Ca: calcium; EPO - erythropoietin, iFGF-23: intact fibroblast growth factor 23; cFGF23: C-terminal fibroblast growth factor 23; PTH: parathyroid hormone; BAP: bone alkaline phosphatase; eGFR: estimated glomerular filtration rate; BMI: body mass index; SD: standard deviation; Q1: lower quartile; Q3: upper quartile.

The inclusion criteria were as follows: CKD stage 3–5 ND (NKF/KDOQI) and Hb <11 g/dL identified in at least two consecutive measurements in the preceding three months, together with transferrin saturation (TSAT) <30%. Any patients with a history of kidney transplantation and who already started dialysis therapy, or those receiving iron supplementation or ESA therapy any time in the previous four weeks were excluded. Other exclusion criteria comprised age <18 years, diabetes mellitus, history of intolerance of intravenous iron supplementation, active inflammation, liver diseases, and mental illness. Basic laboratory tests and a detailed physical examination were carried out at the screening visit. Each patient received five intravenous iron infusions containing the saccharated ferric oxide complex (Vifor France SA) for five consecutive days. Each infusion contained 100 mg of iron, administrated as a 100 mL vial of 0.9% iron (III)-hydroxide sucrose complex in NaCl; in addition, 24-h intervals were left between each 40-min intravenous infusion. The participants remained hospitalized for the duration of the study. During this time, the subjects received a standardized diet prepared by a hospital dietician containing 750 mg phosphorus and 700 mg calcium per day and were instructed not to eat any other food. On the first and third day immediately before the administration of the iron infusion, and two hours afterwards, blood samples were collected for the measurement of the following blood parameters: c-FGF23 and iFGF23, PTH, bone alkaline phosphatase (BAP), phosphorus (PO4), calcium (Ca), erythropoietin (EPO). All the measurements were repeated on day 6. After collection, the blood samples were immediately centrifuged at 3000 rpm at 4°C for 10 min, and the supernatants were frozen at −80°C  until analysis. Plasma iFGF23 and cFGF23 were measured by immunosorbent essay (ELISA) (intact FGF-23, Human 2^nd^ generation and cFGF-23, 2^nd^ generation, TECO medical, Sissach, Switzerland). Bone-specific alkaline phosphatase was measured in serum with ELISA (TECO medical group, Sissach, Switzerland). Plasma erythropoietin was measured with ELISA (Erythropoietin Human TECO medical, Sissach, Switzerland).

Serum high-sensitive CRP calcium, phosphorus, creatinine, iron, ferritin, total iron-binding capacity (TIBC), plasma PTH and hemoglobin were measured using standard automated laboratory methods.

### Statistical analysis

All statistical analyses were performed and graphs plotted using Statistica version 13.1 PL software. The distribution of the continuous variables was assessed with the Shapiro-Wilk test. Depending on the normality of distribution, the variables were presented as mean ± standard deviation or median [lower quartile, upper quartile]. The repeated measures ANOVA or Friedman ANOVA were used for comparing multiple dependent groups, with subsequent *post hoc* tests. The Mann-Whitney U-test was performed to compare two independent groups. Spearman’s correlation coefficient was calculated for non-parametric correlations. A p-value of <0.05 was considered statistically significant.

## Results

**Figure 1.**
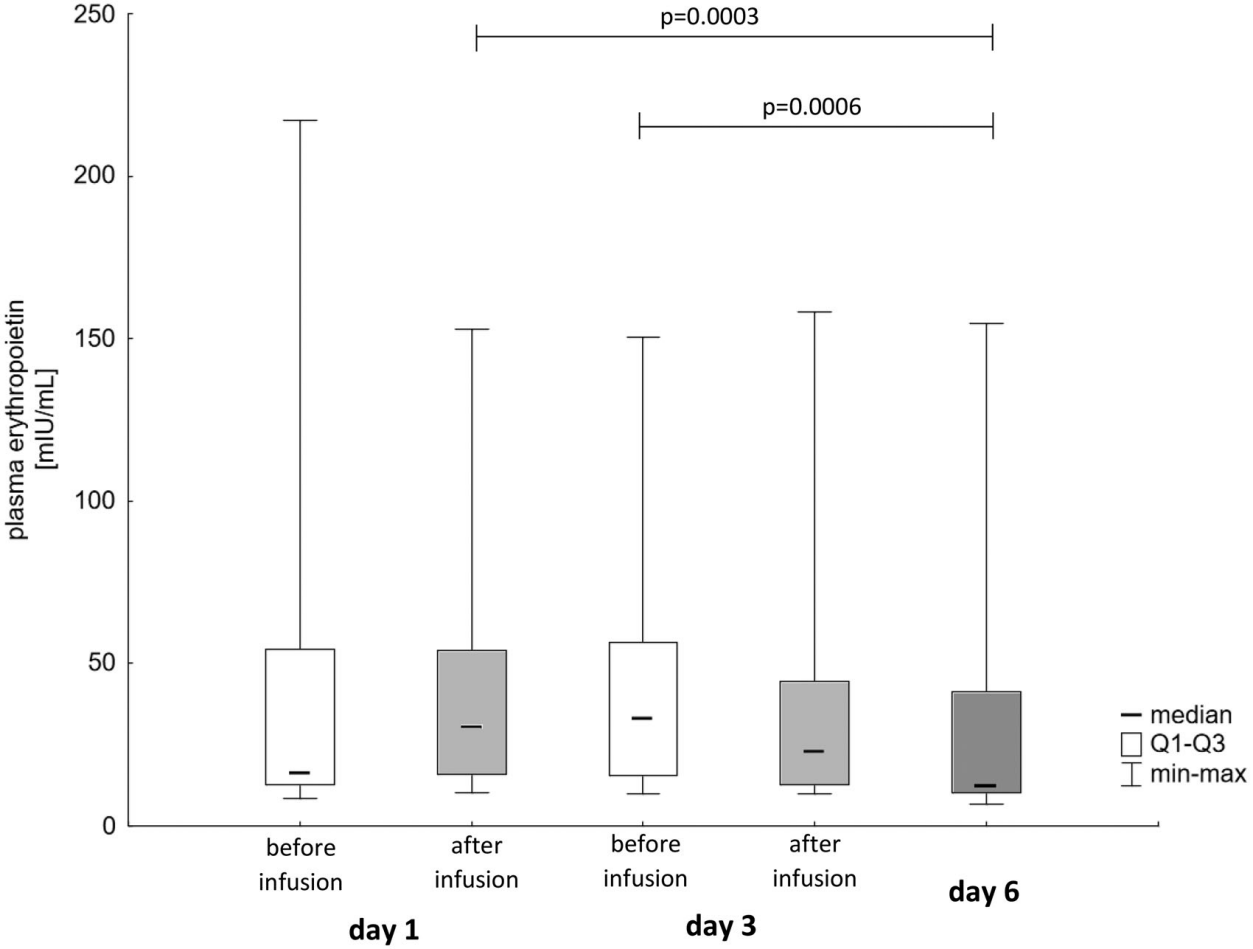
Plasma EPO - erythropoietin concentrations during the study (Friedman ANOVA *p* = 0.00015, *p*-values of significant *post hoc* comparisons provided).

The plasma EPO concentration was significantly lower at the end of the iron treatment than two hours after the first iron infusion (*p* = 0.0003) and before the third iron dose (*p* = 0.0006) (median [Q1, Q3]: 12.6 [10.2, 41.4] mIU/mL. vs. 30.9 [15.9, 54.2] mIU/mL and 33.4 [15.4, 56.7] mIU/mL, respectively). All plasma EPO values assessed during the study are shown in Figure 1.

**Figure 2.**
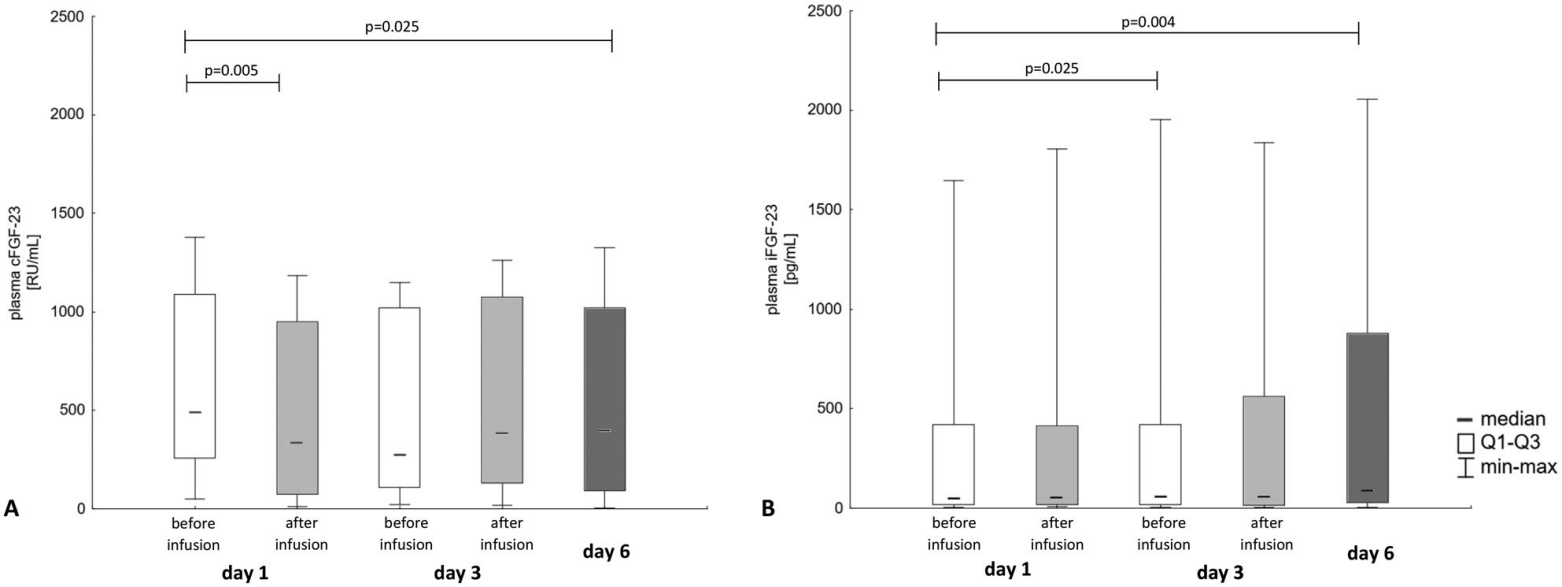
Plasma cFGF-23 – C-terminal fibroblast growth factor 23 (A) and iGF-23-intact fibroblast growth factor 23 (B) concentrations during the study (Friedman ANOVA *p* = 0.0052 and *p* = 0.0021, respectively; *p*-values of significant *post hoc* comparisons provided).

Conversely, plasma iFGF-23 increased significantly before the third dose (61.1 [18.6, 420.1] pg/mL; *p* = 0.025) and at the end of the treatment (92.1 [28.4, 878.1] pg/mL; *p* = 0.004) compared to the pretreatment value (48.4 [16.2, 420] pg/mL). cFGF-23 concentration decreased significantly after the first iron dose (491.8 [257.7, 1086.3] vs. 339.2 [75.4, 951.2] RU/mL; *p* = 0.005) and after treatment completion (398.7 [90.4, 1022.3]) vs. baseline (*p* = 0.025). The changes of FGF-23 concentrations during the study are shown in Figure 2.

No significant linear correlation was found between changes in EPO and FGF-23 (iFGF-23 and cFGF-23) during the study from baseline to day 6 (delta values). Nor were any observed for plasma EPO and FGF-23 changes in the first (baseline vs. 2 h after iron infusion on day 1) and the third day (before vs. 2 h after iron infusion on day 3) of the treatment.

Regarding the individual data, in 16 of the 35 patients (45.7%), the increase of iFGF-23 concentration was accompanied by a decrease of EPO concentration.

**Figure 3.**
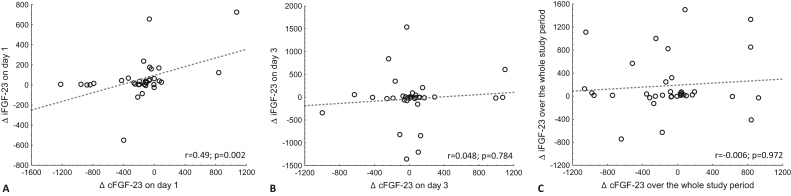
Correlations between changes in plasma iFGF-23 - intact fibroblast growth factor 23 and cFGF-23 - C-terminal fibroblast growth factor 23 concentrations on day 1 (A, before vs. 2 h after iron infusion on day 1), on day 3 (B, before vs. 2 h after iron infusion on day 3) and over whole period of study (C, baseline vs. day 6). Spearman test applied for all analyses.

A positive, significant linear correlation was found between the changes in iFGF-23 and cFGF-23 on the first day of treatment (before treatment vs. 2 h after iron infusion on day 1). However, this effect was not observed on the third day (before vs. 2 h after iron infusion on day 3) nor throughout the treatment period (baseline vs. day 6), as presented in Figure 3.

**Figure 4.**
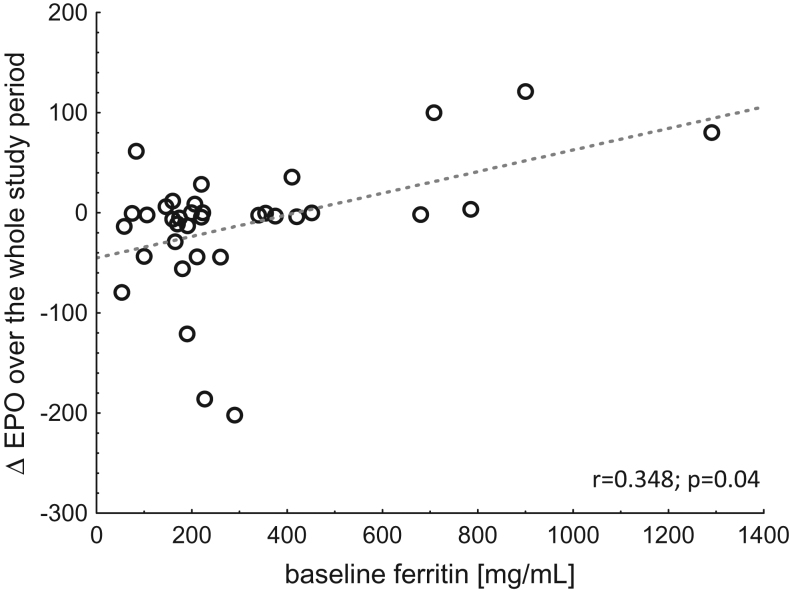
Correlation between changes in plasma EPO - erythropoietin concentrations over the whole study period (baseline vs. day 6) and baseline ferritin concentration, Spearman’s test applied.

The changes in EPO (baseline vs. day 6) correlated significantly with baseline ferritin concentration (Figure 4).

Moreover, concentrations of calcium, phosphate, PTH and BAP at the different time points of the study are shown in Table 2.

**Table 2.** Concentrations of calcium, phosphate, PTH and BAP at the different time points of the study.

Parameter	Day 1		Day 3		Day 6	*p* value
	before infusion	after infusion	before infusion	after infusion		
Ca [mmol/L] mean ± SD	2.00 ± 0.26	1.95 ± 0.28	2.07 ± 0.25	2.06 ± 0.35	2.02 ± 0.31	0.0898^a^
Phosphate [mmol/L] median [Q1, Q3]	1.59 [1.35, 1.8]	1.57 [1.3, 1.78]	1.69 [1.32, 1.89]	1.61 [1.35, 1.82]	1.53 [1.29, 1.92]	0.0529^b^
PTH [pg/mL] median [Q1, Q3]	3.51 [1.2, 21.08]	4.61 [1.45, 30.56]*	7.5 [1.3, 24.86]	3.66 [0.52, 13.21]	2.49 [0.62, 10.75]	**0.0434^b^**
BAP [U/L] median [Q1, Q3]	22.9 [19.3, 27.4]	23.1 [19.1, 28.3]	21.6 [19, 28.9]	22.4 [18.3, 28.1]	24.5 [20.6, 30.9]	0.3560^b^

Repeated measures ANOVA (a) was applied for calcium comparisons and Friedman ANOVA (b) for phosphate, PTH and BAP comparisons. Bold values are statistically significant at *p* < 0.05.

*p < 0.05 compared with day 6. PTH: parathormone; BAP: bone alkaline phosphatase; SD: standard deviation; Q1: lower quartile; Q3: upper quartile.

## Discussion

The main finding of the study was that administration of iron hydroxide sucrose complex IV infusion in non-dialysis CKD patients led to a parallel increase of plasma iFGF23 and a decrease of endogenous erythropoietin concentration.

The links between endogenous erythropoietin production and mineral metabolism have not been thoroughly explored. However, in an experimental study, Coe et al. [12] found that plasma erythropoietin levels were significantly reduced in mice treated with recombinant FGF23; however, they also report that a loss of FGF-23 resulted in increased erythropoiesis in both peripheral blood and bone marrow [12].

These findings suggest that a link exists between FGF23 secretion and renal anemia, independent of the severity of disturbances of other markers of mineral metabolism. In addition, some changes in mineral metabolism were observed in the CKD patients; for example, an increase in cFGF23 increase was noted during iron infusion, followed by a decrease in serum erythropoietin concentration.

Although many studies have investigated the role of the phosphatonin FGF-23 in the pathogenesis of CKD, only a few have assessed its possible contribution to the development of renal anemia. However, a large-scale prospective cohort study found that high serum FGF23 levels were associated with an increased risk of anemia in patients with non-dialysis CKD [13].

One of the considered theories was that inflammation may be one of the mediators of these changes. Interestingly, David et al. found that chronic inflammation increased biologically active iFGF23 levels [14] and might also reduce the activity of FGF23 cleavage enzymes or enhance post-translational O-glycosylation of FGF23 by GALNT3, which protected FGF23 from cleavage [14–16].

Although the role of FGF-23 has been thoroughly investigated in the last two decades, the laboratory method of serum FGF-23 measurement remains a subject of controversy [17]. That may arise from the fact that two different commercial ELISAs are available for the determination of FGF-23 in human plasma. Both methods were used in the present study, as they allowed the examination of both intact FGF23, which detects the full-length FGF23 molecule, and C-terminal FGF-23, which detects both intact and C-terminal FGF-23 fragments. cFGF-23 was selected for this study as it has has less biological variability than iFGF-23 [17].

Another important fact was that, in our study blood was taken in the morning each day to avoid a potential influence of diurnal fluctuations in serum FGF23 and EPO levels [17]. In addition, the patients stayed in a hospital room for the entire duration of the study, which allowed the drug infusions to be performed at exactly the same time each day. The phosphate and calcium content of the diet was also controlled during the treatment.

It was decided to only include patients with moderate or severe renal impairment who were not yet on dialysis. While FGF-23 plasma concentration always increases in the early stages of kidney disease, patients with end-stage kidney disease who have started hemodialysis demonstrated a much more pronounded increase in plasma FGF-23 than expected for the degree of kidney dysfunction. Therefore, the analysis of such data would have been significantly impaired [18].

In our study group, the highest increase of FGF23 concentration was observed after the first three days of the treatment, which was consistent with the results of previous studies that showed the strongest effect on mineral metabolism to occur up to 72 h after the commencement of iron administration [19].

As mentioned above, iron supplementation alone can induce an inflammatory response that may impair endogenous erythropoietin production. The presence of low-grade inflammation in most CKD patients could interfere with this effect. Therefore, patients with greatly increased CRP at baseline, which suggested acute inflammation, were excluded from the study [20].

In addition to the presence of an inflammatory state, the type of iron preparation may also matter, since previous studies indicated that the chemical structure of the iron compound used for iv therapy might influence treatment-induced changes of mineral metabolism and probably secondary epo changes [21,22]. It was found that the intravenous iron sucrose infusion was associated with the most marked hypophosphatemia and osteomalacia [21,22]. This may be because the FGF-23 receptors present on osteocytes interact with iron [23]; to avoid this effect, the present study used iron hydroxide III sucrose [23,24].

Another important fact was that parallel changes in FGF-23 and EPO concentration were observed during the study; however, unlike animal studies, our results were not significant [25]. Similarly, Gravsen et al. found that neither iron isomaltoside or ferric carboxymaltose infusions had any effect on plasma levels of iFGF23 and phosphate for up to seven days of the treatment [26]. Other possible interactions between the EPO and FGF-23 signaling pathways were noted by Daryadel et al. and Clinkenbeard et al. who emphasize the role played by erythroid progenitor cells with FGF receptors, which was one of the main connecting factor between EPO and mineral metabolism [25,27].

A noteworthy study examined whether exogenous EPO stimulates FGF23 *in vivo*. The findings indicate that both cFGF23 and iFGF23 increased in four patients after EPO administration, but no such effect was observed in patients who underwent kidney transplantation. In that group, higher cFGF23 concentration was noted in those with higher serum EPO concentration, and this relationship was independent of graft function [27]. Many studies performed in animal models, with and without CKD, found a much higher concentration of cFGF23 after the infusion of recombinant EPO, but only a small increase of iFGF-23 [25,26,27,28].

However, studies based on FGF23-knockout mice or mice injected with iFGF23-blocking peptide resulted in increased erythropoiesis, reduced erythroid cell apoptosis and elevated renal and bone marrow erythropoietin mRNA expression with increased levels of circulating erythropoietin [29].

An association has found between FGF23 and erythropoiesis in animal studies; however, this was suppressed at higher FGF-23 concentrations. In addition, all mentioned relationships appeared to be bi-directional, since elevated FGF-23 may reduce bone marrow EPO expression, and EPO may promote FGF-23 transcription in turn [8,30]. The detailed mechanism remains unclear but seems to be independent of iron, which contrasts with our hypothesis and findings.

In our study we were not able to find any significant correlation between EPO and FGF-23 changes during the study period. No correlation was observed regarding changes in iFGF nor cFGF; however, by analyzing the individual data, it was found that in about half of the patients, the increase in iFGF-23 concentration was accompanied by a parallel decrease in EPO concentration.

The duration of iron therapy in the present study was six days, which seemed to be sufficient to explore the potential effect of an increased FGF23 concentration on endogenous EPO production. However, the changes in EPO and stimulation of erythropoiesis appear to be slow-acting processes [31].

Our study has several limitations. The number of patients was low; however, it should be noted that there have only a scarce evidence coming from experimental study in this field [20,29]. As such, the present work should only be treated as a preliminary study, and further, larger studies are required to confirm our findings. In addition, further research should examine the levels of inflammatory cytokines to exclude the possibility that inflammation impairs EPO production.

In conclusion, intravenous iron administration significantly affects mineral metabolism in non-dialysis CKD patients, but may also independently suppress the secretion of endogenous erythropoietin. Our findings may have implications for the diagnosis and treatment of renal anemia.
